# Immunity evasion: consequence of the N501Y mutation of the SARS-CoV-2 spike glycoprotein

**DOI:** 10.1186/s43141-021-00287-z

**Published:** 2022-01-14

**Authors:** Henrietta Onyinye Uzoeto, Judith Nnedimkpa Ajima, Amarachukwu Vivian Arazu, Glory Omini Ibiang, Samuel Cosmas, Olanrewaju Ayodeji Durojaye

**Affiliations:** 1 Federal College of Dental Technology, Enugu, Enugu State Nigeria; 2 Department of Biological Sciences, Coal City University, Emene, Enugu State Nigeria; 3 Department of Chemical Sciences, Coal City University, Emene, Enugu State Nigeria; 4 Department of Science Laboratory Technology, University of Nigeria, Nsukka, Enugu State Nigeria; 5 Department of Biochemistry, University of Nigeria, Nsukka, Enugu State Nigeria; 6 Department of Molecular and Cell Biology, School of Life Sciences, University of Science and Technology of China, Hefei, China

## To the Editor,

### Main text

The trimeric spike glycoprotein-mediated entry of the SARS-CoV-2 (severe acute respiratory syndrome coronavirus 2) is the first step of the replication cycle of the virus. The mature spike trimer of the SARS-CoV-2 is made up of the S1 (exterior) and S2 (transmembrane) subunits. Attachment is mediated by the S1 subunit for its interaction with the ACE2 (angiotensin-converting enzyme 2) of the host protein, while the transmembrane subunit facilitates the fusion between the cellular and viral membranes. Hence, the spike glycoprotein plays a crucial role in the replication process of the virus. It is likewise a protein of interest due its immunogenicity and its abundance, both at viral surface and the surface of infected cells [1].

As of early February 2021, over a hundred million people have been infected worldwide by the SARS-CoV-2 and more than two million deaths linked to the same virus (https://covid19.who.int). SARS-CoV-2 as a member of the coronavirus family carries the most abundant genome among single-stranded RNA viruses. Some SARS-CoV-2 spike glycoprotein mutations have been shown to increase the viral infectivity. An example of such mutations is the D614G spike glycoprotein mutation which was shown to increase the infectivity of the virus by 8- to 10-folds in susceptible cells [2]. Moreover, both the transmissibility and infectivity of the D614G mutant virus have been reported to be significantly increased in a hamster model [3]. Fortunately, the 614G mutation did not result in any significant alteration in the antigenicity of the virus that may facilitate an escape from the immune response that results from vaccination or infection with the original virus [4].

All viruses mutate over time. Many of the changes have little or no effect on the viral properties. However, some mutations may impact the properties of the virus, such as the severity of associated diseases, the rate of spread, the performance of therapeutic drugs, vaccines, diagnostic tools, social, and other public health measures [5]. As of July 2021, no regular naming for the SARS-CoV-2 variants has been established. Most organizations, including news agencies and governments, describe variants by the countries where the first set of infection cases were identified. Following several months of discussion, Greek letter nomenclature was announced by the World Health Organization on the 31st of May 2021 for the important viral strains. By this, variants can be described in a fashion that will completely eradicate stigmatization as a result of the use of country names [5]. COVID-19 patients have been reported to experience various symptoms, ranging from mild to a serious sickness. Examples of such symptoms are fever, breathing difficulties or shortness of breath, fatigue, muscle aches, headache, loss of smell and taste, sore throat, nose congestion, nausea, and diarrhea [6].

The lineage B.1.1.7 recently named the Alpha variant is a SARS-CoV-2 variant. As one of the many variants of concern, it is estimated that the transmissibility of the variant is about 80% more than that of the wild-type. The first detection of the Alpha variant was in November 2020 in the UK, and a quick spread was experienced around mid-December as the infection surged [7]. Among the several mutations of the variant is the N501Y substitution, which occurs in the receptor-binding motif (RBM) of the spike protein receptor-binding domain (RBD) [7]. The lineage B.1.351 recently named the Beta variant is also a SARS-CoV-2 variant known to be a variant of concern. The variant was discovered first in South Africa around October 2020 in the Nelson Mandela Bay metropolitan area of the Eastern Cape province [8]. Among the several mutations of this variant are the receptor-binding domain mutations (K417N, E484K, and N501Y), two of which occur in the receptor-binding motif of the virus (E484K and N501Y) [8]. The lineage P.1 recently named the Gamma variant is another variant of the SARS-CoV-2. It was discovered on the 6th of January 2021 by the NIID (National Institute of Infectious Diseases) in Tokyo and was labeled as a Variant of Concern by the World Health Organization thereafter [9]. Similar to the Beta variant of the virus, the Gamma variant possesses both the E484K and N501Y mutation in its receptor-binding motif, but differs in its K417 substitution, where a threonine (T) replaces lysine (K) [9]. The B.1.617.2 is the most recent SARS-CoV-2 Variant of Concern to emerge. This variant is termed the Delta variant and was first detected in India, and has since spread rapidly internationally. The Delta variant was labeled as a Variant of Concern by British scientists on the 6th of May 2021 after evidence were flagged that it spreads as fast as the Alpha variant and more rapidly than the wild-type version of the virus [10]. The Delta variant mutates two residues in its receptor-binding domain that are different from the RBD-mutations of other variants (L452R and T478K). Both mutations have also been reported to occur in the receptor-binding motif of the spike protein [10].

**Fig. 1:**
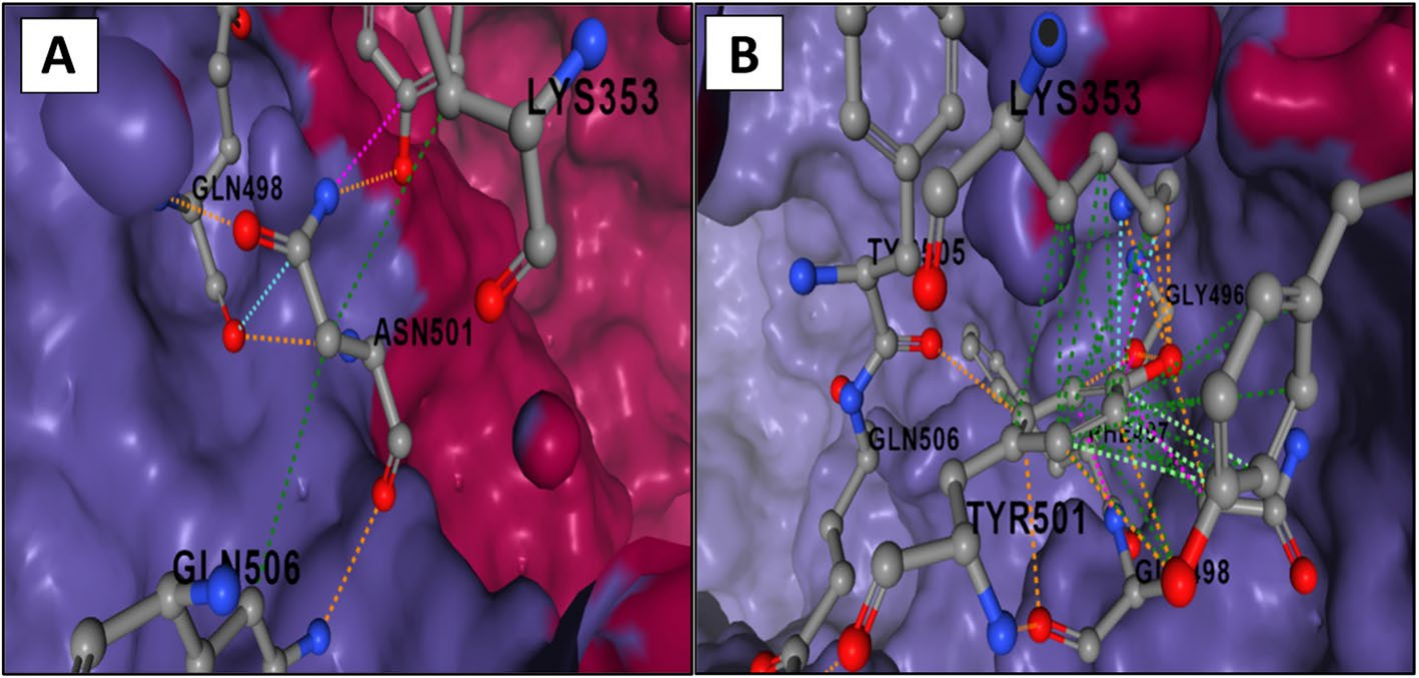
Surface representation of the interaction interface between the **a** wild-type SARS-CoV-2 spike glycoprotein receptor-binding domain (RBD) and the human angiotensin-converting enzyme (hACE-2). **b** Interaction interface between the N501Y mutant SARS-CoV-2 spike glycoprotein RBD and the hACE-2. The blue and red-colored surfaces represent the SARS-CoV-2 spike glycoprotein RBD and the hACE-2 binding interfaces, respectively. The pink, cyan, red, green, and orange colored lines represent the clash, Van der Waals, hydrogen, hydrophobic, and polar bonds, respectively

An important review has been attempted by Nandi et al. It was focused that the receptor-binding domain (RBD) protein of SARS-CoV-2 spike and the angiotensin-converting enzyme 2 (ACE2) of the host receptor interact, and further replication of coronavirus spike protein causes its invasion in the host cell. The human lymphocyte antigen 6 complex, Locus E (LY6E) inhibits the entry of CoV into host cells by interfering with the human gene through the spike protein-mediated membrane fusion. Some natural formulations have also been shown to prevent the spike protein from binding to the host cell [11]. Results from a recent study by Li et al. [12] showed that the SARS-CoV-2 501Y.V2 variants do not confer increased viral infectivity in multiple cell types, except for the ACE2-overexpressing cells of murine, where a significant increase in infectivity was observed. Notably in this study [12], the 501Y.V2 variants susceptibility to twelve out of seventeen neutralizing monoclonal antibodies was substantially diminished. The neutralization ability of the sera from immunized mice and convalescent patients also diminished significantly for these variants. The observed resistance to monoclonal antibody neutralization was majorly caused by the N501Y mutation at the receptor-binding domain of the spike glycoprotein. The increased infectivity that was observed in the ACE2-overexpressing cells of murine suggests the possibility of a spillover of the 501Y.V2 variants to mice [12]. Following this line of study, we conducted a computational analysis using the Arpeggio visualizer [13], to examine the changes in interatomic interactions that occurred upon the single mutation of the N501 residue of the SARS-CoV-2 spike glycoprotein to tyrosine (Y) (PDB 6LZG). The implementation of Arpeggio is in Python, and it utilizes OpenBabel and BioPython for the processing of structure files in PDB format. OpenBabel is used for the assignment of atom types to all atoms in the PDB structure through SMARTS queries (a molecular language for pattern matching), while the implementation of the KDTree in BioPython is used for the extraction of nearest-neighbor atoms in the cutoff range of 5 angstroms. Every pairwise interatomic contact is assigned a SIFt (structural interaction fingerprint) using an extended definition of the types of interaction. The first 5 bits of the fingerprint depict if the interaction is proximal, van der Waals’, van der Waals’ clashing, covalent bond, or steric clash, and all are mutually exclusive. The first 4 bits are synchronized based on the theoretical van der Waals’ and covalent radii that are defined in OpenBabel. The remaining interactions in the 5-angstrom cutoff range are proximal, although they may not denote a meaningful interaction. The remaining bits correspond to specific feature interactions, such as carbonyl, hydrophobe–hydrophobe, metal complex, and aromatic interactions, also ionic, halogen, and hydrogen bonds [13]. Consistent with Li et al. experimental results, we observed an increased non-covalent protein-protein interaction network upon the N501Y mutation (Fig. 1), suggesting an immunity escape by the virus.

Based on this observation, we sought further to understand the immunity escape mechanism through the computational analysis of the existing interaction between the wild-type and mutant spike protein-antibody complex. Biocytogen recently designed RenMab (a novel human antibody mouse) in which the whole human antibody variable region was used to replace the 3.2 megabase kappa chain and 2.6 megabase heavy chain of the entire variable region in the mouse model, while constant regions remain unmodified (www.renmab.com). Upon several foreign antigen immunizations, the mouse model was observed to have mounted vigorous antibody responses. Nie et al. [14] in a recent study on the RenMab mouse models screened antibodies binding specifically to the receptor-binding domain of the SARS-CoV-2 upon immunization and discovered interaction with the human ACE2 was efficiently inhibited. The antibodies were reported to bind the spike protein receptor-binding domain with high affinity (within the nanomolar range) and likewise displayed appreciable neutralizing properties against pseudotyped viruses. However, despite the exhibited neutralization properties of the antibodies, recent mutations in the SARS-CoV-2 spike glycoprotein have been reported to have high chances of evading existing preventive therapies [14].

**Fig. 2:**
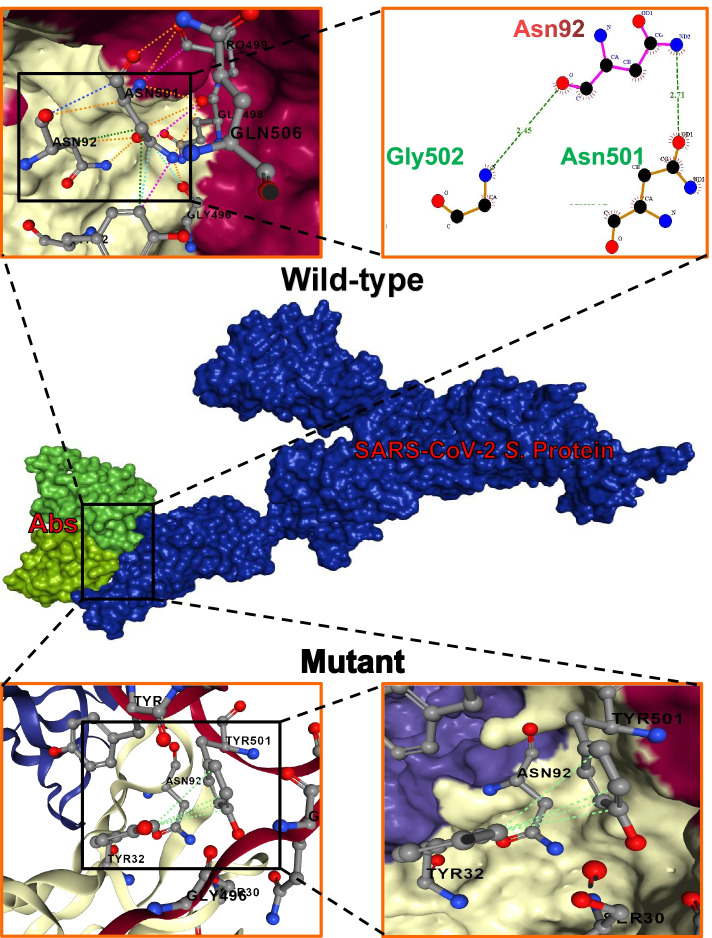
Interface analysis of the wild-type and mutant SARS-CoV-2 spike glycoprotein upon the docking of antibody against the RBD. The mid-segment shows a surface representation of the protein-antibody interaction, with the 3D structure of the full-length SARS-CoV-2 spike glycoprotein coloured in blue, and both chains of the antibody colored in different shades of green. The top and bottom segments show details of the observed changes in the interaction between the wild-type and mutant spike glycoprotein upon the N501Y mutation

Using the HDOCK tool [15], we defined the specific antibody-binding region of interest by highlighting the N501 and other surrounding residues, after which the Ab4 neutralizing antibody dimer (PDB 7E39) was docked against spike protein (PDB 6XCN). HDOCK is an extensively integrated tool for the management and incorporation of biological information for fast and robust protein-protein docking, structure prediction, homology search, and template-based molecular modeling [15]. Having successfully completed the docking protocol, the protein-protein interaction was analyzed using Arpeggio [13], supported with the LigPlot software [16] to visualize the interaction surface in 2D. The interaction analysis output showed several interatomic interactions between the receptor-binding domain residues of the wild-type SARS-CoV-2 and the bound neutralizing antibody, such as hydrophobic (green), polar (orange), carbonyl (blue), and Van der Waals (cyan) interactions (Fig. 2). Upon the N501Y mutation, the observed interatomic interactions with the wild-type viral protein were replaced with aromatic bonds with the adjacent TYR32 residue of the neutralizing antibody. We therefore hypothesize that the virus mutates this N501 residue to escape antibody neutralization through the loss of important interatomic interactions, while the same mutation resulted in an increased interaction with the human ACE2.

## Conclusion

Mutations in the receptor recognition site of the SAR-CoV-2 spike glycoprotein has been explained through recent findings as a strategy by the virus to evade neutralizing antibodies rather than increasing the viral infectivity. The observed increase in non-covalent interactions, as observed in our computational study also gives credence to the experimental report, by revealing a more complex interaction network, which speculatively translates into an increased binding affinity of the viral RBD to the human ACE2 receptor protein. The revealed atomic details from the interaction analysis in this study can serve as a useful tool for antibody engineering towards a more efficient antiviral therapeutics.

## Data Availability

Not applicable

## Abbreviations

SARS-CoV-2: Severe acute respiratory syndrome coronavirus 2
COVID-19: Coronavirus disease 19
ACE2: Angiotensin converting enzyme 2
RBD: Receptor-binding domain
